# The Hedgehog Signaling Networks in Lung Cancer: The Mechanisms and Roles in Tumor Progression and Implications for Cancer Therapy

**DOI:** 10.1155/2016/7969286

**Published:** 2016-12-26

**Authors:** Yoshinori Abe, Nobuyuki Tanaka

**Affiliations:** Department of Molecular Oncology, Institute for Advanced Medical Sciences, Nippon Medical School, 1-396 Kosugi-cho, Nakahara-ku, Kawasaki 211-8533, Japan

## Abstract

Lung cancer is the most common cause of cancer-related death worldwide and is classified into small cell lung cancer (SCLC) and non-small-cell lung cancer (NSCLC). Several gene mutations that contribute to aberrant cell proliferation have been identified in lung adenocarcinoma, a part of NSCLC. Various anticancer drugs that target these mutated molecules have been developed for NSCLC treatment. However, although molecularly targeted drugs are initially effective for patients, the 5-year survival rate remains low because of tumor relapse. Therefore, more effective drugs for lung cancer treatment should be developed. The hedgehog (HH) signaling pathway contributes to organ development and stem cell maintenance, and aberrant activation of this signaling pathway is observed in various cancers including lung cancer. In lung cancer, HH signaling pathway upregulates cancer cell proliferation and maintains cancer stem cells as well as cancer-associated fibroblasts (CAFs). Furthermore, physical contact between CAFs and NSCLC cells induces HH signaling pathway activation in NSCLC cells to enhance their metastatic potential. Therefore, HH signaling pathway inhibitors could be a useful option for lung cancer therapy.

## 1. Introduction

**Figure 1:**
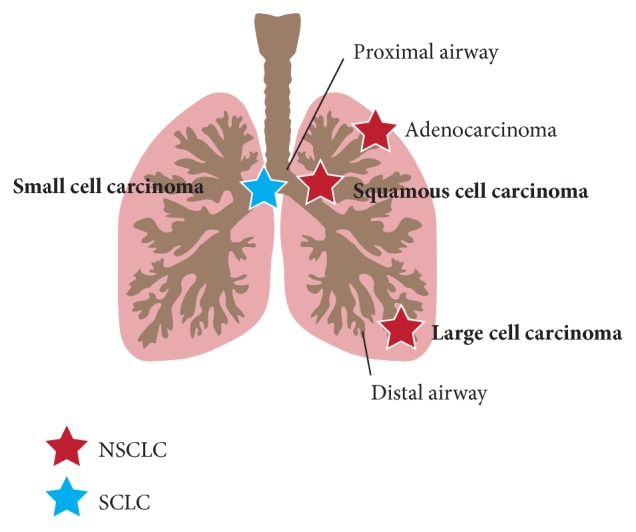
Lung cancer. Lung cancer is mainly classified into small cell lung cancer (SCLC) and non-small-cell lung cancer (NSCLC). NSCLC is further classified into adenocarcinoma, squamous cell carcinoma, and large cell carcinoma. Adenocarcinoma is the most common lung cancer and arises in the distal airway. Squamous cell carcinoma and SCLC arise in the proximal airway. Large cell carcinoma also arises in the distal airway.

Lung cancer is a leading cause of cancer-related death worldwide [1]. Lung cancer is classified into two major types: small cell lung cancer (SCLC) and non-small-cell lung cancer (NSCLC) (Figure 1). SCLC arises in the midlevel airway and is a very aggressive, highly metastasizing and lethal cancer type that comprises 15% of all lung cancers. NSCLC is the major type of lung cancer and comprises 85% of all lung cancers. NSCLC includes lung adenocarcinoma, lung squamous cell carcinoma (LSCC), and lung large cell carcinoma. Adenocarcinoma arises in the distal airway and its incidence is not related to smoking. LSCC arises in the proximal airway and is more aggressively and strongly associated with smoking than adenocarcinoma. Large cell carcinoma arises in the distal airway and the cancer cell mass is larger than the other two types of NSCLC. Large cell carcinoma is also an aggressive tumor [2]. Despite our current understanding of lung cancer, the precise molecular mechanisms underlying tumorigenesis in the lung have still not been completely determined.

Several signaling pathways are aberrantly activated in lung cancer cells. Key oncogenic mutations, so-called driver mutations, in components of these signaling pathways have been identified in lung adenocarcinoma. These include epidermal growth factor receptor* (EGFR)*, the Kirsten rat sarcoma viral oncogene homolog GTPase* (KRAS)*, a member of the rapidly accelerated fibrosarcoma (RAF) family, B-RAF* (BRAF)*, and the fusion oncogene echinoderm microtubule-associated protein-like 4-anaplastic lymphoma receptor tyrosine kinase* (EML4-ALK)* [3, 4]. Furthermore, gene amplifications of avian erythroblastic leukemia viral oncogene homolog 2* (ERBB2)*,* MET*,* ROS1*, Neuregulin 1* (NRG1)*, neurotrophic tyrosine kinase receptor 1* (NTRK1)*, and* RET* have also been found in lung adenocarcinoma [5–8]. In LSCC, discoidin domain-containing receptor 2* (DDR2)*, fibroblast growth factor receptor 1* (FGFR1)*,* FGFR2*, and* FGFR3* and genes in the phosphatidylinositol 3-kinase (PI3K) pathway seem to be more commonly mutated [9]. These gene mutations and gene amplifications induce activation of signaling pathways related to cell proliferation, such as the Ras-extracellular signal-regulated kinase (ERK) pathway and the signal transducer and activator of transcription 3 (STAT3) pathway. NSCLCs harboring* EGFR* mutations or* ALK* gene rearrangements have been successfully targeted with tyrosine kinase inhibitors (TKIs) [10, 11]. However, these TKIs have not yet been shown to improve the overall survival in patients because of tumor recurrence [12]. Moreover, there are no effective drugs for SCLC, LSCC, and large cell carcinoma. Therefore, the 5-year survival rate of lung cancer is only 16% at present [1].

A number of morphogenic signaling pathways that regulate developmental processes and organ homeostasis play critical roles in lung tumorigenesis. Studies of cancer stem cells (CSCs) support the idea that tumors harbor hallmarks of early development in their gene expression repertoire [13]. Recently, remarkable findings from an early stage clinical trial of an inhibitor for the hedgehog (HH) signaling pathway have renewed hope that disruption of developmental signaling in tumors can be of therapeutic benefit [14, 15]. HH pathway inhibitors block both intrinsic signaling in cancer cells and extrinsic signaling to stromal cells to reduce tumor growth [16]. These two strategies exploit distinct oncogenic functions of the pathway. As the HH signaling pathway is activated in SCLC as well as NSCLC, HH pathway inhibitors are expected to become a useful tool for treatment of lung cancer.

In this review, we discuss the roles of the HH signaling pathway in tumor development in SCLC and NSCLC and components of the HH signaling pathway that represent viable lung cancer therapy targets.

## 2. The HH Signaling Pathway

**Figure 2:**
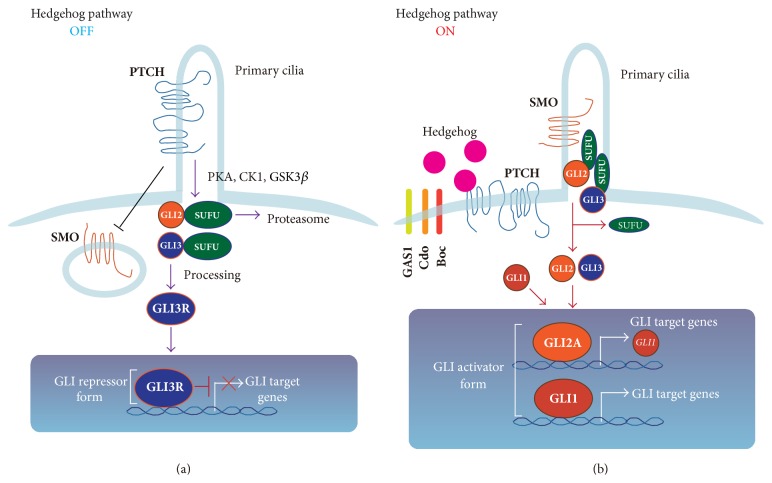
HH signaling pathway during development. (a) In the absence of HH ligands, PTCH blocks ciliary localization of SMO and the GLI repressor form (mainly GLI3 repressor form [GLI3R]) suppresses induction of GLI target gene expression. (b) In the presence of HH ligands, the HH pathway is activated. Binding of HH ligand to PTCH prevents PTCH inhibition of SMO, and SMO is free to translocate into primary cilia and is fully activated. SMO then activates the GLI family, mainly GLI2. GLI2 upregulates expression of* GLI1* as well as GLI target genes. GLI1 is also activated downstream of SMO. Activated GLI2 (GLI2A) and GLI1 further upregulate expression of various GLI target genes.

The HH signaling pathway regulates morphogenesis of various organs during embryogenesis [17]. The HH signaling pathway also regulates stem cell renewal and organ homeostasis in the adult [18]. The molecular mechanisms of the HH pathway are complex, and several comprehensive reviews have been published describing the detailed mechanisms [19–21]. In the canonical HH signaling pathway, three HH ligands have been identified: Sonic Hedgehog (SHH), Indian Hedgehog (IHH), and Desert Hedgehog (DHH). Each HH ligand has distinct spatial and temporal expression patterns and activates HH signaling by binding to Patched (PTCH), a 12-pass transmembrane-spanning receptor. In the absence of HH ligand, PTCH is localized to primary cilia and constitutively suppresses the activity of Smoothened (SMO), a 7-pass transmembrane-spanning protein, which is a member of the G-protein-coupled receptor superfamily [22] (Figure 2). In addition to PTCH, additional HH ligands binding cell surface proteins, such as CAM-related/downregulated by oncogenes (CDO), brother of Cdo (BOC), and growth-arrest-specific 1 (GAS1), have been identified, and these molecules function as HH ligand coreceptors to facilitate HH signal reception [23, 24]. Following binding of one of the three HH ligands to PTCH, SMO accumulates in the primary cilia and facilitates the activation of GLI transcriptional activators and their translocation into the nucleus to activate expression of HH target genes, including* GLI1* and* PTCH* genes (Figure 2) [25, 26]. Suppressor of fused (SUFU) is a key negative regulator of the HH signaling pathway [27]. In the absence of HH ligands, SUFU inhibits HH signaling by sequestration of GLI proteins in the cytoplasm and by promoting the formation of the GLI3 repressor (GLI3R). A nuclear function for SUFU in chromatin has also been suggested.

In vertebrates, the GLI family consists of three proteins, GLI1, GLI2, and GLI3 [21]. All GLI proteins contain an activator domain (GLI-A) at their C-terminus; GLI2 and GLI3 also have an N-terminal repressor domain (GLI-R) [28]. Studies in mutant mice suggest that GLI2 is the major activator of HH signaling pathway [29], whereas GLI3 is the major repressor [30, 31]. GLI1 most likely serves as a signal amplifier downstream of GLI2 [29, 32].* Gli2* knockout (KO) mice die at birth, whereas* Gli1* KO mice show normal development, unless one copy of* Gli2* is also defective [33]. Interestingly, experiments in mutant mice further suggest that GLI2 can rescue GLI1 protein function, whereas* Gli1* knock-in into the* Gli2* allele can rescue the* Gli2* null phenotype [34]. Upon binding of the HH ligand to the receptor PTCH, followed by SMO activation, SUFU-GLI2 and SUFU-GLI3 complexes dissociate and GLI2 and GLI3 translocate into the nucleus, where they activate expression of HH target genes, including GLI1 and PTCH [35]. The balance between the activating and repressive forms of the GLI family transcription factors results in the expression of target genes [21].

The HH signaling pathway has critical roles during embryonic lung development as well as postnatal lung development [36]. During embryonic lung development, HH signaling pathway molecules dramatically change expression patterns and expression levels. The SHH expression pattern from embryonic day (E) 10 to 16.5 is important for branching and growing bronchi [37]. After E16.5, SHH expression is restricted to a subset of the epithelial cells [37]. PTCH expression pattern in growing bronchi mirrors the expression pattern of SHH [38]. PTCH is also expressed in mesenchyme around E11.5 [39]. Smo is reportedly expressed in epithelium and mesenchyme between E12.5 and E16.5 (pseudoglandular stage) [40]. GLI1, GLI2, and GLI3 are expressed in the mesenchyme during the pseudoglandular stage, and their levels decrease near birth [41]. Although SHH and PTCH expression levels are decreased at birth, they are still observed in epithelial cells [38]. Reduction of the HH signaling pathway in the postnatal lung induces abnormal lung maturation. Therefore, the HH signaling pathway is also involved in postnatal lung maturation [42, 43]. In the healthy adult lung, HH signaling maintains adult lung quiescence and regulates repair [44]. However, it is still currently unclear how HH signaling can promote quiescence on the one hand and tumorigenesis on the other.

**Figure 3:**
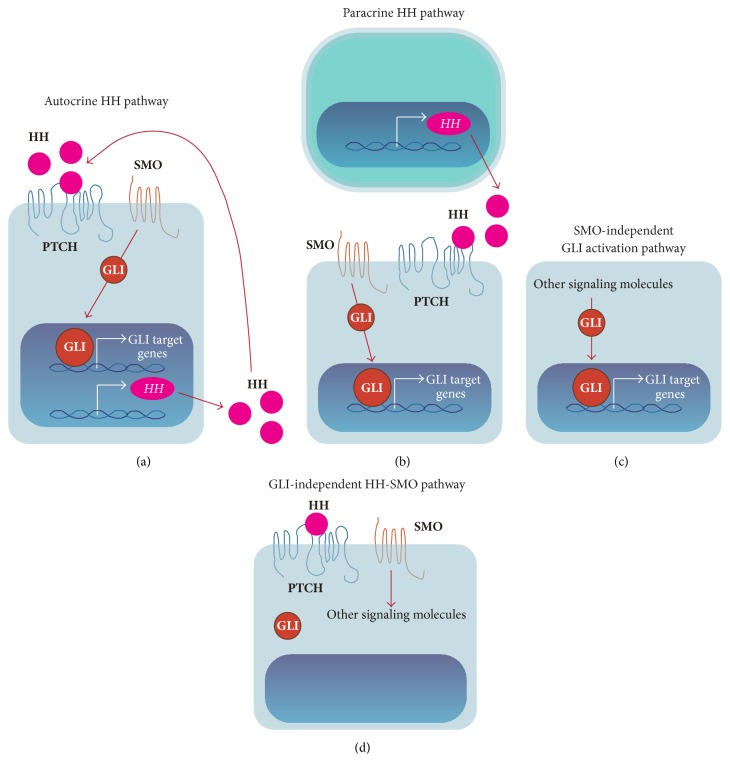
Modes of HH-GLI signaling pathway activation in cancer. (a) HH ligands constitutively activate the HH pathway in HH ligand-producing cancer cells by autocrine signaling. Cancer cells produce HH ligand, and secreted HH ligands activate the HH signaling pathway in these cancer cells. (b) Cancer cell-mediated production of HH ligands also activates the HH pathway in stroma cells (e.g., CAFs) to maintain cancer cells by paracrine signaling. (c) GLI family transcription factors are activated in a SMO-independent manner, called the noncanonical pathway. (d) SMO can also activate other signaling molecules in cancer cells.

Constitutive activation of HH signaling has been observed in many cancers (e.g., skin, lung, stomach, and colon) [45] and promotes cancer cell proliferation, metastasis, and CSC maintenance. Multiple mechanisms of HH signaling pathway activation in cancer have been proposed. Somatic mutations in HH pathway components and overproduction of HH ligands cause aberrant HH signaling pathway activation. Somatic mutations of* PTCH1* and* SMO* were identified in patients with basal cell carcinoma and medulloblastoma [46–49]. Other mutations in genes encoding HH pathway components have been reported, including* SUFU* in medulloblastoma [50] and* GLI1* and* GLI3* in pancreatic adenocarcinoma [51]. Moreover,* GLI1* amplification was observed in glioblastoma [52]. HH ligand overproduction was observed in upper gastrointestinal tract, pancreas, colon, and metastatic prostate cancers, as well as SCLC, glioblastomas, and melanomas [53–58]. Overproduction of HH ligands constitutively activates the HH pathway in HH ligand-producing cancer cells by autocrine signaling [53, 54] and in stroma cells such as cancer-associated fibroblasts (CAFs) surrounding HH ligand-producing cancer cells by paracrine signaling [16, 59] (Figure 3). In addition, noncanonical HH signaling has been defined as ligand-dependent activation of SMO but independent of GLI activation [60] or as GLI activation independent of SMO. The noncanonical GLI activation pathway includes transforming growth factor *β* (TGF-*β*) [61], EGFR [62], Ras-Erk [63, 64], and PI3K-Akt-mechanistic target of rapamycin (mTOR) [65] signaling pathways.

## 3. HH Signaling Pathway in SCLC

Although mutation or amplification of genes involved in the HH pathway has not been found in SCLC, the HH signaling pathway was activated in many SCLC cases [66]. Watkins et al. found HH pathway activation in neuroendocrine cells in later lung development (E16.5) and the airway epithelium during repair of acute airway injury [53]. Neuroendocrine cells are considered candidates for the origin of SCLC. HH pathway activation was also observed in SCLC tissue and this observation was confirmed by analysis of SCLC cell lines. Moreover, a SCLC cell xenograft model using nude mice demonstrated that the HH pathway was activated in SHH-producing SCLC cells but not in surrounding non-SHH-producing cancer cells, suggesting that HH pathway activation was an autocrine and/or juxtacrine loop in SCLC. Analysis using a SCLC model mouse also revealed that HH pathway activation initiated and progressed mouse SCLC independent of the tumor microenvironment. Furthermore, suppression of SMO in a SCLC mouse model strongly suppressed initiation and progression of SCLC [67]. In addition, immunohistochemistry analysis revealed upregulation of HH pathway components in SCLC patients, suggesting that the HH signaling pathway is also activated in SCLC patients [68].

A recent study reported a novel crosstalk between the HH pathway and bombesin- (BBS-) like neuropeptide-mediated signaling in SCLC [69]. SCLC cells secrete BBS, which acts as an autocrine growth factor. Expression of both SHH and gastrin-releasing peptide receptor (GRPR), a BBS-cognate receptor, was observed in 56% of SCLC. Analysis of SCLC cell lines revealed that BBS signaling activates GLI1 activity and that BBS-mediated GLI1 activation is suppressed by cyclopamine, a SMO inhibitor. Furthermore, GLI1 activation was mediated by BBS signaling-nuclear factor-*κ*B- (NF-*κ*B-) mediated production of SHH ligand in SCLC cells.

## 4. HH Signaling Pathway in NSCLC

Various studies have also demonstrated that the HH pathway is activated in NSCLC. The expressions of GLI1 target genes, such as Forkhead Box M1* (FOXM1)*, B cell-specific Moloney murine leukemia virus integration site 1* (BMI1)*, and* NANOG*, were elevated in NSCLC patients [70, 71]. Another study showed that 40 S ribosomal protein S6 kinase 2 (p70S6K2) regulates GLI1 activity in NSCLC cells. siRNA-mediated p70S6K2 knockdown suppressed cell viability and GLI1 activity, and p70S6K2 knockdown promoted GLI1 degradation through inhibition of glycogen synthase kinase 3*β*- (GSK3*β*-) mediated GLI1 phosphorylation. However, a SMO inhibitor, 3-keto-N-aminoethylaminocaproyldihydrocinnamoyl- (KAAD-) cyclopamine [72], did not affect GLI1 activity, and PI3K inhibitor treatment suppressed GLI1 activity [73].

CAFs are widely defined as all fibroblast cells within the tumor stroma and key players in the process of tumorigenesis through modulation of tumor microenvironment, CSC maintenance, and regulation of tumor metabolism [74]. CAF proliferation is maintained by various factors such as growth factors (e.g., TGF-*β* and platelet-derived growth factor [PDGF]) and cytokines (e.g., interleukin 1 [IL-1] and IL-6) [75]. Bermudez et al. showed that NSCLC cells can secrete SHH ligand, and secreted SHH ligand activates the HH signaling pathway in CAFs. This pathway activation induces CAF proliferation [76]. Huang et al. showed that PTCH, SMO, and GLI2 expressions were upregulated in LSCC-derived cell lines. However, SMO inhibitor treatment or SMO knockdown demonstrated only a minor inhibitory effect on cell proliferation, whereas GLI2 suppression significantly suppressed cell proliferation and induced extensive apoptosis. Therefore, GLI transcriptional activity would be regulated by a noncanonical (SMO-independent) pathway [77]. These reports suggest that the HH pathway is activated by the paracrine mechanism and GLI activation in NSCLC cells is regulated by the noncanonical (SMO-independent) pathway.

On the other hand, several studies have reported that HH signaling is activated by the autocrine pathway in NSCLC cells. The aggressiveness of NSCLC has been shown to be associated with the acquisition of epithelial-to-mesenchymal transition (EMT) [78]. A549 lung adenocarcinoma cells that obtain mesenchymal phenotype (A549-M cells) show upregulated SHH ligand and GLI1 expression compared with A549 cells. In A549-M cells, the HH pathway was activated by autocrine signaling, and suppression of the HH pathway contributed to suppression of TGF-*β* signaling-induced cancer cell migration and metastatic characteristics [79].

CAFs can secrete various growth factors and cytokines. Secreted proteins induce extracellular matrix (ECM) remodeling. Furthermore, CAFs interacts with cancer cells and CAF-secreted proteins activate various signaling pathway by paracrine signaling. ECM remodeling and CAFs-mediated paracrine signaling pathway activation could induce metastatic properties of cancer cells [75]. Choe et al. [80] showed that EMT-related gene expression and the HH signaling pathway was upregulated in adenocarcinoma cells by means of direct coculture of NSCLC cells and lung CAFs. The authors proposed that metastatic properties might be acquired by direct interaction of adenocarcinoma cells and CAFs and CAF-mediated paracrine HH signaling pathway activation in adenocarcinoma cells.

CSCs exhibit a self-renewing capacity and are responsible for tumor maintenance and relapse [81]. CSC maintenance in adenocarcinoma and LSCC are regulated by the autocrine HH signaling pathway. Several molecules and enzymatic activities such as CD44, CD133, and high aldehyde dehydrogenase (ALDH) activity have been identified as CSC markers of NSCLC [82–85]. The HH signaling pathway was activated in CD44^high^/ALDH^high^ cancer cells harboring CSC properties [86]. Furthermore, CD133^+^ NSCLC cells also exhibit CSC properties and secrete SHH ligand, and HH pathway inhibition in CD133^+^ cells attenuated sphere formation, suggesting that the autocrine HH pathway is involved in CD133^+^ CSC maintenance [87]. Although CD133^+^ SCLC cells are identified as a CSC phenotype [88], there is no evidence that the HH pathway is involved in SCLC stem cell maintenance. Moreover, a previous report showed that GLI1 upregulated expression of the embryonic stem cell transcription factor SRY- (sex determining region Y-) box 2 (SOX2) by cooperation with EGF signaling in lung adenocarcinoma-derived cell lines [89]. As described above, the interaction of CAFs and NSCLC cells induces metastatic properties of NSCLC cells via CAF-mediated HH signaling pathway activation in NSCLC cells. Chen et al. showed that an interaction of CAFs and NSCLC cells and CAF-mediated HH signaling pathway activation in NSCLC cells are also involved in CSC maintenance [90]. We independently observed that GLI1 inhibition but not SMO inhibition attenuated sphere formation, suggesting that GLI1 activity was regulated by other signaling pathways for NSCLC stem cell maintenance (unpublished data).

SOX2 expression is upregulated in LSCC [91], and therefore SOX2 is used as one of the tumor markers for LSCC. Although SOX2 has critical roles in CSC maintenance, the precise mechanism of SOX2-mediated CSC maintenance is largely unknown. Justilien et al. reported that the SOX2-HH pathway has important roles for CSC maintenance in LSCC. Protein kinase C iota (PRKCI) phosphorylated Ser394 in SOX2, resulting in upregulated expression of hedgehog acyltransferase* (HHAT)*. The SHH ligand is changed to its active form by HHAT, resulting in HH signaling pathway activation. The PRKCI-SOX2-HH signaling pathway plays important roles in CSC maintenance [92].

As described above, SMO inhibitor treatment suppressed EMT properties through remodeling of the actin cytoskeleton and motility of NSCLC cells [79]. Although SMO inhibition downregulated EMT-associated gene expression, expressions of GLI1 target genes were not affected [93]. These results suggest that SMO might activate other signaling molecules as well as GLI transcription factors in NSCLC cells harboring mesenchymal properties.

Many studies on the roles of the HH signaling pathway in NSCLC suggested that GLI1 and GLI2 play central roles in tumor progression, tumor metastasis, and CSC maintenance. The mechanisms of GLI activation are diverse in cancer cell types and the tumor microenvironment surrounding cancer cells, since GLI is activated by various pathways including the autocrine and paracrine HH pathways as well as canonical and noncanonical GLI activation pathway.

## 5. HH Signaling Pathway-Targeted Cancer Therapy in Lung Cancer

Previous studies have revealed that subsets of lung cancer patients harbor mutations in the key oncogenic drivers upon which tumor survival and progression are dependent. These include mutations in EGFR and the EML4-ALK fusion protein [3]. Therefore, various TKIs targeting EGFR and EML4-ALK have been developed. However, the clinical efficacy of TKIs differs among patients, and acquired resistance for chronic treatment often develops in most patients who are treated with TKIs [5, 94, 95]. Furthermore, there are no effective anticancer drugs for SCLC, LSCC, and large cell carcinoma.

Previous studies reported that tumor volume and tumor recurrence were suppressed by HH pathway inhibitor treatment or combination treatment of HH pathway inhibitors and other types of chemotherapeutic agents such as TKIs and platinum-containing drugs. Park et al. [67] demonstrated that combination treatment of etoposide and a SMO inhibitor (LDE225: Sonidegib) [96] attenuated tumor recurrence of SCLC using a mouse xenograft model. Moreover, LDE225 treatment attenuated the TKI-resistant NSCLC cell line HCC827-GR (gefitinib resistant) derived tumor growth. In addition, cotreatment of SMO inhibitor and MET inhibitor to HCC827-GR xenografted tumors further suppressed tumor volume, since constitutive MET activation was observed in HCC827-GR cells [97]. Moreover, RNAi-mediated GLI1 knockdown suppressed tumor formation and tumor sphere formation. Several SMO inhibitors and GLI inhibitors have been developed [14]. GDC-0449 (Vismodegib) [98] is approved for basal cell carcinoma therapy, and several SMO inhibitors including GDC-0449 are used in clinical investigations for SCLC. GLI inhibitors such as GLI-antagonist- (GANT-) 58, GANT-61, HH pathway inhibitor- (HPI-) 1, Genistein, and Glabrescione B (GlaB) have also been developed [43, 99–101]. In addition, arsenic trioxide (ATO), which suppresses GLI1 transcriptional activity [102, 103], is used in clinical investigations as a GLI inhibitor (Table 1) [14]. However, other GLI inhibitors have not yet progressed to clinical trials. Since the HH pathway and GLI activity have important roles in lung cancer formation and lung CSC maintenance, these chemical compounds may be useful for lung cancer therapy.

## 6. Conclusion

**Figure 4:**
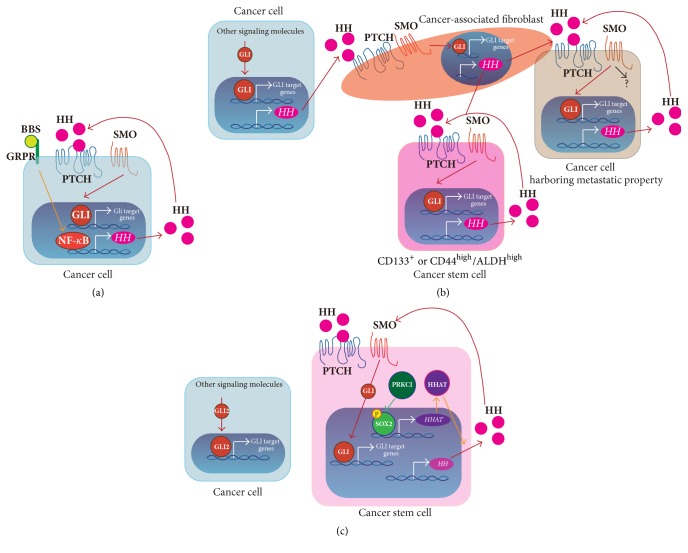
The role of the HH signaling pathway in lung cancer. (a) The HH signaling pathway in SCLC. The autocrine HH signaling pathway promotes cancer cell proliferation. (b) The HH signaling pathway in adenocarcinoma. The noncanonical GLI activation pathway would maintain cancer cell proliferation. CAF maintenance would be regulated by paracrine HH signaling pathway activation. CAF-secreted HH ligands would activate the HH signaling pathway in cancer cells and CSCs. CAFs-mediated paracrine HH pathway activation in cancer cells has important roles in acquisition of metastatic properties. Moreover, CAF-mediated HH signaling pathway activation might be involved in CSC maintenance. Cancer cells harboring metastatic properties and CSCs would be also maintained by autocrine HH signaling pathway activation. In addition, SMO might activate other signaling molecules in cancer cells harboring metastatic properties. (c) The HH signaling pathway in LSCC. Cancer cells would be maintained by the noncanonical GLI2 activation pathway. PRKCI-SOX2-HH signaling pathway has important roles in CSC maintenance.

We have discussed the relationship between the HH signaling pathway and lung cancer and the mechanism of HH signaling pathway activation in lung cancer. As summarized in Figure 4, the GLI activation machinery and the role of the HH pathway in lung cancer are different in NSCLC and SCLC as well as among the types of NSCLC. Furthermore, the HH signaling pathway is involved in the interaction of cancer cells and CAFs for tumor maintenance. Various SMO inhibitors are used in clinical investigations for lung cancer. Results from* in vitro* and* in vivo* experiments have demonstrated that SMO inhibitor treatment is effective for lung tumor suppression. In fact, SMO inhibitors are used in clinical trials for SCLC. The HH signaling pathway is involved in CSC maintenance, tumor progression, and metastasis in NSCLC. Therefore, SMO inhibitors may be a better option for lung cancer therapy in the future. However, previous studies suggest that GLI transcription factors are activated by various mechanisms, including the SMO-independent pathway. In particular, dysregulated SMO-independent GLI activation pathway may cause SMO inhibitor resistance. Several GLI inhibitors have also been recently developed. Therefore, a HH-pathway-activated lung cancer therapy using GLI inhibitors would be an effective option. To develop the most effective HH pathway inhibitor for treatment of lung cancer, the current challenge is not only to accelerate HH inhibitor development but also to more deeply understand the regulatory mechanism of GLI-mediated transcription.

## Figures and Tables

**Table 1:** The HH signaling pathway inhibitors.

Inhibitor Name	Organization	Clinical Trial
(1) SMO inhibitors		
Cyclopamine, KAAD-cyclopamine	—	No
GDC-0449 (Vismodegib/Erivedge)	Roche/Genentech/Curis	Yes (phases 0, I, and II)
LDE225 (Erismodegib/Sonidegib/Odomzo)	Novartis	Yes (phases 0, I, and II)
BMS-833923/XL139	Bristol Myers Squibb/Exelixis	Yes (phases I and II)
PF-04449913 (Glasdegib)	Pfizer	Yes (Phase II)
PF-527857	Pfizer	No
LY2940680 (Taladegib)	Ignyta	Yes (phases I and II)
IPI-926 (Sadegib)	Infinity	Yes (phase I)
TAK-441	—	No
MRT-92	—	No
(2) GLI inhibitors		
GANT-58, GANT-61	—	No
Arsenic trioxide (ATO)	—	Yes (phases I, II, III, and IV)
HPI-1	—	No
Glabrescione B (GlaB)	—	No

See [14] for description of the clinical trials of HH signaling pathway inhibitors.
